# Impact of portal hypertension on short‐ and long‐term outcomes after liver resection for intrahepatic cholangiocarcinoma: A propensity score matching analysis

**DOI:** 10.1002/cam4.4222

**Published:** 2021-08-18

**Authors:** Jun Fu, Qinjunjie Chen, Yuyan Yu, Wuyi You, Zongren Ding, Yuzhen Gao, Haitao Li, Yongyi Zeng

**Affiliations:** ^1^ Department of Hepatopancreatobiliary Surgery Mengchao Hepatobiliary Hospital of Fujian Medical University Fuzhou China; ^2^ The Big Data Institute of Southeast Hepatobiliary Health Information Fuzhou China; ^3^ Department of Hepatic Surgery IV The Eastern Hepatobiliary Surgery Hospital Naval Medical University Shanghai China; ^4^ Department of Radiology Imaging Mengchao Hepatobiliary Hospital of Fujian Medical University Fuzhou China; ^5^ Department of Clinical Laboratory Sir Run Run Shaw Hospital, Zhejiang University School of Medicine Hangzhou China

**Keywords:** hepatectomy, liver cancer, portal hypertension, prognosis, propensity score matching

## Abstract

**Objective:**

We explored the impact of clinically significant portal hypertension (CSPH) on short‐ and long‐term outcomes of intrahepatic cholangiocarcinoma (ICC) after liver resection (LR).

**Methods:**

Data of 352 ICC patients with cirrhosis who underwent LR were extracted from the Primary Liver Cancer Big Data (PLCBD) between 2005 and 2015 and reviewed. A nomogram based on logistic analyses was developed to illustrate the influencing factors of post‐hepatectomy liver failure (PHLF). The impact of CSPH on long‐term survival was explored through propensity score matching (PSM) analysis, log‐rank test, Cox proportional hazards model, and Kaplan–Meier curves.

**Results:**

A total of 106 patients had CSPH, and 246 patients did not. A nomogram established based on GGT level, CSPH, intraoperative blood loss, and multiple tumors had an area under the receiver operating characteristic curve of 0.721 (95% confidence interval [CI] = 0.630–0.812), which displayed a better PHLF predictive value than the MELD score (0.639, 95% CI = 0.532–0.747) and Child–Pugh score (0.612, 95% CI = 0.506–0.719). Moreover, the patients with CSPH had worse overall survival (OS) rates than the patients without CSPH in the whole cohort (*p *= 0.011) and PSM cohort (*p *= 0.017). After PSM, multivariable Cox analyses identified that CSPH was an independent risk factor for OS (hazard ratio = 1.585, 95% CI = 1.107–2.269; *p *= 0.012).

**Conclusion:**

CSPH is a significant risk factor for PHLF and OS in ICC patients with cirrhosis after surgery. Selecting the proper patients before operation can effectively avoid PHLF and improve the prognosis of ICC.

## Graphical abstract

The present study sought to examine the influence of clinically significant portal hypertension (CSPH) on short‐term survival, especially the occurrence of post‐hepatectomy liver failure in intrahepatic cholangiocarcinoma (ICC) after surgery. In addition, we used the propensity score matching analysis to explore the impact of CSPH on long‐term survival in ICC patients.

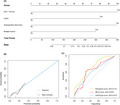

## 1 INTRODUCTION

Intrahepatic cholangiocarcinoma (ICC), the second leading primary liver cancer, has been increasing in incidence worldwide during the past decades, accounting for 5%–20% of the primary liver cancer population.^1^, ^2^ Although the exact pathogenesis of ICC remains unclear, current studies have demonstrated that multiple causative risk factors, including hepatitis virus infection, hepatolithiasis, and cirrhosis, are involved in the development of this malignancy.^3^, ^4^ Cirrhosis can be observed in 27.8%–50.5% of patients with ICC,^5^, ^6^, ^7^ which is largely a result of viral hepatitis, alcohol abuse, and hepatolithiasis.^8^

Liver resection (LR) remains the potential curative therapy for patients with ICC.^9^ However, many challenges remain in the management of LR.^10^ On the one hand, the resectability of ICC is still low, and patients with unresectable tumors have an extremely short survival time.^11^ On the other hand, even after LR, the risk of tumor recurrence and metastases is still high, resulting in a poor survival outcome.^12^, ^13^ The currently reported 5‐year overall survival (OS) rates after hepatectomy range from 20% to 35%.^14^, ^15^ The effectiveness of liver transplantation, transarterial chemoembolization, and ablation remains to be determined in patients with ICC.^16^ A recent study has summarized the clinical efficacy of futibatinib, a FGFR antagonist, and pointed out that ICC patients with FGFR2 gene fusions or other rearrangements could acquire a survival benefit from futibatinib.^17^ However, this conclusion needs further studies to be confirmed in the future.

In general, some cirrhotic patients present with clinically significant portal hypertension (CSPH) as time goes by, which is considered a contraindication for surgery. However, LR is also performed in selected oriental patients with CSPH because of a lack of other effective therapies for ICC.^5^, ^6^, ^7^ Although the effectiveness and safety of LR in these patients have been reported,^7^ the impact of CSPH on short‐ and long‐term survival after LR in those patients remains unclear.

This study sought to verify the influence of CSPH on short‐term survival, especially the incidence of post‐hepatectomy liver failure (PHLF), in ICC after surgery. The impact of CSPH on long‐term survival in patients with ICC after hepatectomy was also explored through propensity score matching (PSM).

## 2 PATIENTS AND METHODS

### 2.1 Patient selection

Data on 394 cirrhotic patients who underwent R0 resection with histopathologically confirmed ICC were extracted from the Primary Liver Cancer Big Data (PLCBD) in Fujian province from September 2005 to December 2015 and reviewed. Of these patients, 42 were excluded as follows: 10 for extrahepatic distant metastasis, 4 for receiving preoperative anticancer treatments, 23 for lost to follow‐up, and 5 for incomplete clinical data. The remaining 352 patients were enrolled in the analysis.

### 2.2 Preoperative work‐up and liver resection

Before surgery, the patients were routinely asked for demographic information. Routine laboratory examinations and imaging studies were also tested. Upper gastrointestinal endoscopy or barium enema examination of the upper digestive tract and cardiopulmonary function were also routinely performed. WHO classification criteria are the basis of the clinical diagnosis of ICC.^15^

Surgical indications were as follows: (1) good performance (ECOG score 0–2) without significant heart, kidney, lung, and other important organ diseases; (2) liver function within Child–Pugh grade A or B7, without refractory ascites; (3) tumor localized to the liver segment or lobe with no evidence of distant tumor metastasis and removal of the liver parenchyma with the possibility of maintaining a liver remnant of ≥50% using measurements from preoperative CT/MRI; (4) no history of variceal bleeding, cirrhosis mild to moderate, and esophageal and gastric varices moderate to severe without bleeding tendency, namely, red color signs; and (5) platelet count ≥75 × 10^9^/L.

Detailed surgical procedures were similar to those previously reported.^18^, ^19^ Regional lymphadenectomy was routinely performed for pre‐operatively or intra‐operatively diagnosed ICC. Histopathological examination of the tumor specimens was routinely performed.

### 2.3 Definitions

The presence of preoperative CSPH was defined as the presence of esophagogastric varices or splenomegaly (diameter >12 cm) with a platelet count <100 × 10^9^/L, which is based on the definition of standard surrogate criteria proposed by the Barcelona Clinic Liver Cancer (BCLC) classification.^20^ The MELD score was calculated using the following formula: MELD = (0.957 × Log_e_ [creatinine in mg/dl] + 0.378 × Log_e_ [bilirubin in mg/dl] + 1.12 × Loge [INR] + 0.643) × 10.^21^ PHLF was defined according to the 50–50 criteria: of PT prothrombin time less than 50% and serum TBIL >50 µmol/L on postoperative day 5.^22^ Surgical complications were classified and graded according to the Clavien–Dindo classification.^23^

### 2.4 Follow‐up protocol and endpoints

After hepatectomy, patients were followed up once every 2 months in the first 2 years and once every 3–6 months thereafter. The patients with CSPH were asked if they had any tendency or signs of bleeding, including upper gastrointestinal hemorrhage. The diagnosis of ICC recurrence was the same as that of the initial ICC diagnosis. Treatment options after relapse were discussed by a multidisciplinary team as previously reported.

OS and time to recurrence (TTR) were the primary endpoints of this study. The secondary endpoint was PHLF. OS was calculated as the interval between the date of LR to the date of last follow‐up or patient death. TTR was computed from the date of LR to the date of identification of disease recurrence. Surgical morbidity, including PHLF and mortality, was also observed. This study was censored on 30 October 2018.

### 2.5 Statistical analysis

Continuous variables were described as the median and interquartile range (IQR) or mean ± standard deviation (SD), and Student's *t*‐test or Mann–Whitney *U* test was used to compare differences. Categorical variables were described as the number of cases and percentage, and chi‐squared test or Fisher's exact test was used to compare the differences between the two groups if necessary. A nomogram was established using the “rms” package of R, version 3.1.1 (http://www.r‐project.org/). The performance of the predictive model was assessed using the receiver operating characteristic (ROC) curves and the corresponding area under the curve (AUC). The Kaplan–Meier method was used to plot survival curves, and the log‐rank test was used for comparison. The Cox proportional hazards model was used to analyze independent prognostic factors of OS and tumor recurrence.

A one‐to‐one PSM was used to balance the differences in baseline clinicopathological features between the CSPH and non‐CSPH groups.^24^ Covariates included in the PSM analysis were TBIL, AST, PT, Child–Pugh score, intraoperative blood loss, and tumor number to calculate the propensity score by logistic regression. Individuals in the CSPH group were matched to those in the non‐CSPH group by a 1:1 nearest neighbor matching with a caliper of 0.2 and without replacement to realize the closest estimated propensity score values between the two groups in PSM.^25^ After PSM, a new cohort was created with minimal differences in patient clinicopathological characteristics between the two groups.

Statistical analyses were performed using SPSS (IBM SPSS for Windows, version 22.0; IBM Corp.) and PSM for SPSS version 22.0 (R statistical package version 2.15; Cornell University, USA). All reported *p* values were bilateral, and statistical significance was considered at *p* < 0.05.

## 3 RESULTS

### 3.1 Baseline characteristics

**FIGURE 1:**
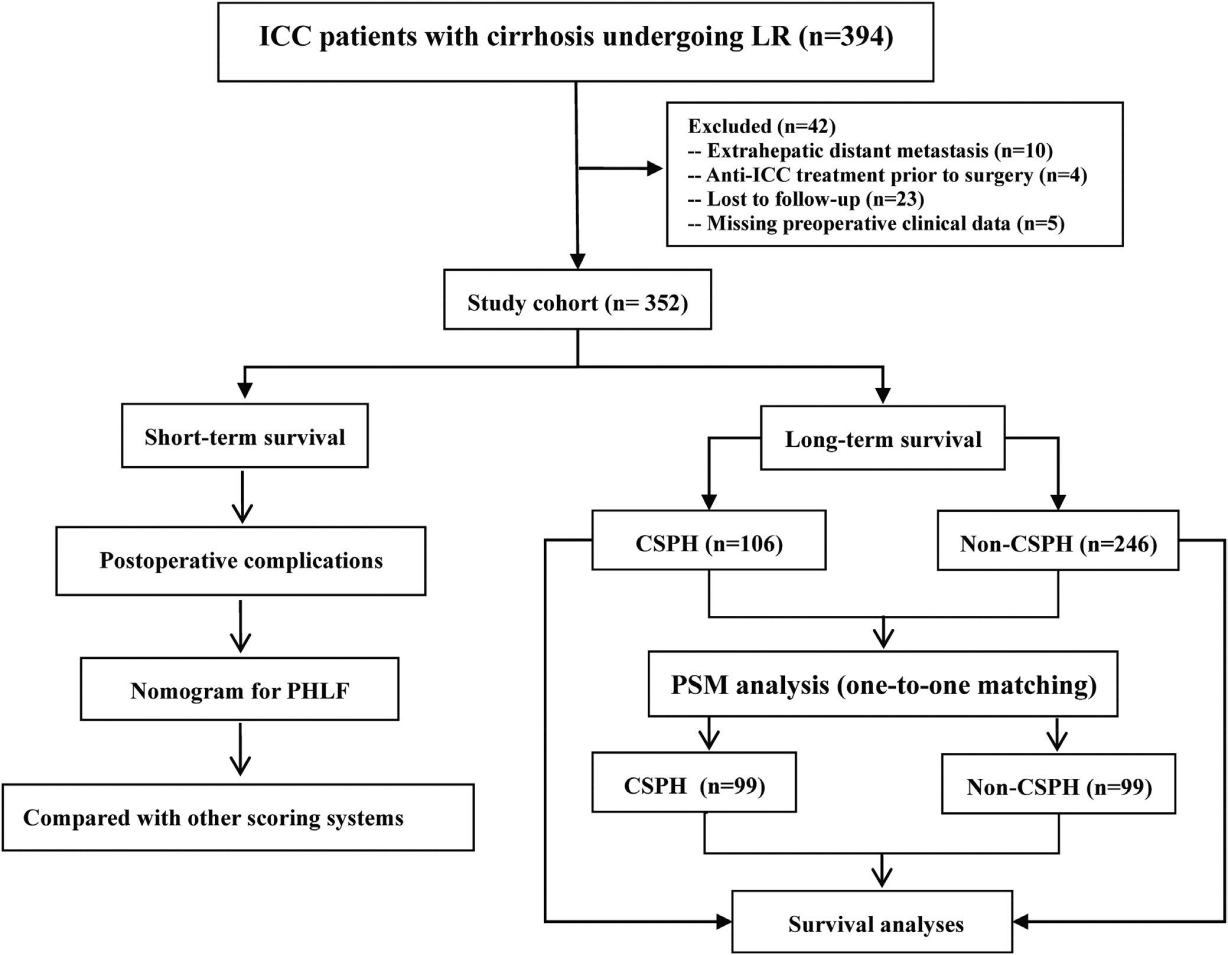
The flow chart of this study

Among 352 patients with cirrhosis who underwent LR, 106 (30.1%) patients had CSPH, whereas the remaining 246 (69.9%) patients did not (Figure 1). Table 1 shows the baseline demographic and clinicopathological characteristics of patients with or without CSPH. After PSM analysis, the baseline characteristics between the two groups were balanced well.

**TABLE 1:** Baseline characteristics for patients with and without CSPH

Variable	Whole cohort (*n* = 352)			PSM cohort (*n* = 198)		
	Non‐CSPH (*n* = 246)	CSPH (*n* = 106)	*p* value	Non‐CSPH (*n* = 99)	CSPH (*n* = 99)	*p* value
Age, year	52.0 ± 10.5	52.7 ± 9.9	0.590	53.7 ± 10.2	53.1 ± 9.9	0.663
Age, >65 years, *n* (%)	29.0 (11.8)	11.0 (10.4)	0.702	16.0 (16.2)	11.0 (11.1)	0.300
Gender, *n* (%)						
Male	209.0 (85.0)	93.0 (87.7)	0.494	83.0 (83.8)	88.0 (88.9)	0.300
Female	37.0 (15.0)	13.0 (12.3)		16.0 (16.2)	11.0 (11.1)	
Hepatobiliary flukes, *n* (%)						
Yes	3.0 (1.2)	3.0 (2.8)	0.371	2.0 (2.0)	3.0 (3.0)	1.000
No	243.0 (98.8)	103.0 (97.2)		97.0 (98.0)	96.0 (97.0)	
Hepatolithiasis, *n* (%)						
Yes	16.0 (6.5)	8.0 (7.5)	0.722	5.0 (5.1)	8.0 (8.1)	0.389
No	230.0 (93.5)	98.0 (92.5)		94.0 (94.9)	91.0 (91.9)	
HBsAg, *n* (%)						
Positive	207.0 (84.1)	92.0 (86.8)	0.524	81.0 (81.8)	85.0 (85.9)	0.440
Negative	39.0 (15.9)	14.0 (13.2)		18.0 (18.2)	14.0 (14.1)	
HCV‐Ab, *n* (%)						
Positive	4.0 (1.6)	3.0 (2.8)	0.434	3.0 (3.0)	3.0 (3.0)	1.000
Negative	242.0 (98.4)	103.0 (97.2)		96.0 (97.0)	96.0 (97.0)	
TBIL, mg/dl^a^	0.8 (0.6, 1.0)	0.9 (0.7, 1.1)	**0.009**	0.8 (0.6, 1.1)	0.9 (0.7, 1.1)	0.238
ALB, g/L	41.3 ± 4.2	41.1 ± 6.1	0.755	40.4 ± 4.6	41.0 ± 5.4	0.381
ALT, IU/L^a^	35.9 (26.3, 55.2)	35.3 (23.7, 57.5)	0.691	35.8 (26.0, 47.9)	35.3 (22.1, 57.6)	0.958
AST, IU/L^a^	32.4 (25.0, 43.6)	32.8 (25.5, 47.2)	0.723	31.9 (24.4, 43.6)	33.4 (26.1, 47.4)	0.456
GGT, IU/L^a^	65.0 (39.0, 131.7)	86.5 (44.0, 168.0)	**0.025**	68.0 (41.0, 116.0)	87.0 (44.0, 168.0)	0.067
PT, second^a^	12.1 (11.7, 12.9)	12.7 (11.8, 13.5)	**0.001**	12.4 (11.7, 13.2)	12.4 (11.7, 13.3)	0.482
Child–Pugh score^a^	5.0 (5.0, 6.0)	6.0 (5.0, 6.0)	**0.003**	5.0 (5.0, 6.0)	5.0 (5.0, 6.0)	0.390
Child–Pugh grade, *n* (%)						
A	229.0 (93.1)	92.0 (86.8)	0.056	92.0 (92.9)	88.0 (88.9)	0.323
B	17.0 (6.9)	14.0 (13.2)		7.0 (7.1)	11.0 (11.1)	
MELD score	7.5 ± 2.0	7.9 ± 2.0	0.077	7.8 ± 2.3	7.8 ± 2.0	0.981
MELD score, *n* (%)						
<9	227.0 (92.3)	90.0 (84.9)	**0.016**	88.0 (88.9)	88.0 (88.9)	0.693
9–10	5.0 (2.0)	9.0 (8.5)		4.0 (4.0)	6.0 (6.1)	
>10	14.0 (5.7)	7.0 (6.6)		7.0 (7.1)	5.0 (5.1)	
AFP, μg/L^a^	7.9 (3.7, 53.5)	9.0 (4.0, 66.7)	0.675	6.4 (3.2, 38.2)	9.4 (3.7, 62.7)	0.313
CEA, μg/L^a^	2.5 (1.6, 4.2)	2.4 (1.6, 3.6)	0.375	2.9 (1.5, 4.4)	2.4 (1.6, 3.7)	0.557
CA19‐9, IU/ml^a^	36.5 (17.1, 103.7)	43.7 (21.5, 84.5)	0.518	41.5 (17.2, 115.8)	43.8 (21.8, 84.0)	0.997
Intraoperative blood loss, *n* (%)						
Yes	37.0 (15.0)	25.0 (23.6)	0.054	18.0 (18.2)	21.0 (21.2)	0.592
No	209.0 (85.0)	81.0 (76.4)		81.0 (81.8)	78.0 (78.8)	
Intraoperative blood loss, ml^a^	200.0 (150.0, 400.0)	200.0 (150.0, 525.0)	0.308	200.0 (150.0, 400.0)	200.0 (150.0, 500.0)	0.742
Hepatectomy, *n* (%)^c^						
Wedge resection	182 (74.0)	89 (84.0)	**0.041**	74 (74.7)	74 (74.7)	0.077
≥1 segment	64 (26.0)	17 (16.0)		25 (25.3)	25 (25.3)	
Pringle maneuver, n (%)						
Yes	202 (82.1)	82 (77.4)	0.300	82 (82.8)	76 (76.8)	0.288
No	44 (17.9)	24 (22.6)		17 (17.2)	23 (23.2)	
Clamp time, minutes^a^	16.0 (9.7, 22.0)	14.5 (5.7, 18.2)	**0.036**	15.0 (8.0, 20.0)	14.0 (5.0, 18.0)	0.177
Operation time, hours^a^	1.7 (1.5, 2.3)	2.0 (1.5, 2.5)	0.053	1.9 (1.5, 2.5)	2.0 (1.5, 2.5)	0.502
Resection margin, cm	0.3 ± 0.6	0.3 ± 0.5	0.243	0.3 ± 0.6	0.3 ± 0.5	0.342
Tumor diameter, cm^a^, ^b^	5.0 (3.7, 7.8)	4.5 (3.2, 8.0)	0.637	4.8 (3.5, 7.3)	4.5 (3.2, 8.0)	0.940
Tumor number, *n* (%)						
Multiple	66.0 (26.8)	15.0 (14.2)	**0.010**	13.0 (13.1)	14.0 (14.1)	0.836
Single	180.0 (73.2)	91.0 (85.8)		86.0 (86.9)	85.0 (85.9)	
Microvascular invasion, *n* (%)						
Yes	40.0 (16.3)	16.0 (15.1)	0.784	17.0 (17.2)	13.0 (13.1)	0.428
No	206.0 (83.7)	90.0 (84.9)		82.0 (82.8)	86.0 (86.9)	
Direct invasion, *n* (%)						
Yes	14.0 (5.7)	6.0 (5.7)	0.991	9.0 (9.1)	5.0 (5.1)	0.267
No	232.0 (94.3)	100.0 (94.3)		90.0 (90.9)	94.0 (94.9)	
Nodal metastasis, *n* (%)						
Yes	51.0 (20.7)	17.0 (16.0)	0.306	21.0 (21.2)	17.0 (17.2)	0.470
No	195.0 (79.3)	89.0 (84.0)		78.0 (78.8)	82.0 (82.8)	

Abbreviations: AFP, alpha‐fetoprotein; ALB, albumin; ALT, alanine transaminase; AST, aspartate aminotransferase; CA19‐9, carbohydrate antigen 19‐9; CEA, carcinoembryonic antigen; CSPH, clinically significant portal hypertension; GGT, gamma‐glutamyl transpeptidase; HBsAg, hepatitis B surface antigen; HCV, hepatitis C virus; INR, international normalized ratio; MELD, model for end‐stage liver disease; PT, prothrombin time; TBIL, total bilirubin.

^a^ Median and IQR; other continuous variables are described in mean ± SD; all categorical variables are reported in percentage (*n* %).

^b^ Largest diameter measure for a solitary tumor; diameter of the largest nodule in multiple tumors.

^c^ A few patients also received splenectomy and/or paraesophagogastric devascularization simultaneously.

Bold values indicate statistical significance.

### 3.2 Surgical complication and PHLF in the whole cohort

Among these 352 patients, 113 (32.1%) patients experienced postoperative complications, with 44 (41.5%) and 69 (28.0%) patients having CSPH or not, respectively (*p *= 0.013). In total, six (1.7%) patients died within 90 days after LR (two from the CSPH group and four from the non‐CSPH group, *p* = 1.000). The hierarchy of Clavien–Dindo complications of grades I/II, III/IV, and V was observed in 33 (31.1%), 9 (8.5%), and 2 (1.9%) and in 51 (20.7%), 14 (5.7%), and 4 (1.6%) from the two groups, respectively.

Among these 352 patients, 27 (7.6%) patients had PHLF, 20 patients presenting with PHLF 90 days after surgery. Another seven patients with PHLF were identified more than 90 days after surgery. Detailed information on complications at 90 days after surgery is listed in Table 2.

**TABLE 2:** Surgical complication in the whole cohort

Complication	Total (*n* = 352)	Non‐CSPH (*n* = 246)	CSPH (*n* = 106)	*p* value
Overall complications, *n* (%)	113 (32.1)	69 (28.0)	44 (41.5)	**0.013**
PHLF, *n* (%)	20 (5.6)	12 (4.8)	8 (7.5)	0.321
Urinary tract infection, *n* (%)	11 (3.1)	6 (2.4)	5 (4.7)	0.260
Wound/fascia dehiscence, *n* (%)	9 (2.6)	5 (2.0)	4 (3.8)	0.342
Refractory ascites, *n* (%)	6 (1.7)	4 (1.6)	2 (1.9)	1.000
Inferior phrenic infection, *n* (%)	6 (1.7)	3 (1.2)	3 (2.8)	0.534
Upper gastrointestinal bleeding, *n* (%)	5 (1.4)	3 (1.2)	2 (1.9)	1.000
Intra‐abdominal hemorrhage, *n* (%)	5 (1.4)	3 (1.2)	2 (1.9)	1.000
Bile leak, *n* (%)	8 (2.3)	6 (2.4)	2 (1.9)	0.750
Pneumonia, *n* (%)	8 (2.3)	5 (2.0)	3 (2.8)	0.645
Pleural effusion, *n* (%)	7 (1.9)	6 (2.4)	1 (0.9)	0.613
Respiratory insufficiency, *n* (%)	5 (1.4)	2 (0.8)	3 (2.8)	0.329
Ileus, *n* (%)	1 (0.3)	0 (0.0)	1 (0.9)	0.664
Liver abscess, *n* (%)	2 (0.6)	1 (0.4)	1 (0.9)	1.000
Cholangitis, *n* (%)	1 (0.3)	0 (0.0)	1 (0.9)	0.664
Hepatic encephalopathy, *n* (%)	1 (0.3)	1 (0.4)	0 (0.0)	1.000
Cerebrovascular accident, *n* (%)	1 (0.3)	1 (0.4)	0 (0.0)	1.000
Arrhythmia, *n* (%)	1 (0.3)	1 (0.4)	0 (0.0)	1.000
Pulmonary embolism, *n* (%)	1 (0.3)	0 (0.0)	1 (0.9)	0.664
Others, *n* (%)	15 (4.2)	10 (3.9)	5 (4.7)	0.781
Clavien grade, *n* (%)				
Grade I and II	84 (23.8)	51 (20.7)	33 (31.1)	0.095
Grade III and IV	23 (6.5)	14 (5.7)	9 (8.5)	
Grade V^a^	6 (1.7)	4 (1.6)	2 (1.9)	

Abbreviations: CSPH, clinically significant portal hypertension; PHLF, postoperative liver failure.

^a^ Grade V: In total, six (1.7%) patients died within 90 days (Grade V). In the non‐CSPH group, three patients died from PHLF without recurrence. One patient without CSPH died of cerebrovascular accident. In the CSPH group, both patients died from PHLF within 90 days without recurrence.

Bold values indicate statistical significance.

### 3.3 Independent risk factors of PHLF

When this study was censored, 27 patients had PHLF, and the occurrence rate of PHLF was 7.6%. Based on the results of multivariable logistic regression analyses, the independent risk factors of PHLF among all 352 patients were GGT >64 IU/L (odds ratio [OR]: 3.477, 95% confidence interval [CI] = 1.414–8.553), CSPH presence (2.711, 1.160–6.335), intraoperative blood loss (3.427, 1.356–8.622), and multiple tumors (2.543, 1.034–6.230) (Table 3).

**TABLE 3:** Uni‐ and multivariable logistic regression analyses of independent risk factors of PHLF in the whole cohort

Variable	Univariable analyses			Multivariable analyses		
	OR	95% CI	*p* value	OR	95% CI	*p* value
Age, Year, >65 versus ≤65	0.604	0.138–2.653	0.504			
Gender, Male versus Female	0.707	0.255–1.963	0.506			
Hepatobiliary flukes, Yes versus No	2.462	0.277–21.861	0.419			
Hepatolithiasis, Yes versus No	0.505	0.066–3.891	0.512			
HBsAg, Positive versus Negative	0.471	0.189–1.177	0.107			
HCV‐Ab, Positive versus Negative	NA	NA	NA			
TBIL, mg/dl, >1.0 versus ≤1.0	2.089	0.941–4.635	**0.070**			
ALB, g/L, <35 versus ≥35	1.158	0.257–5.225	0.849			
ALT, IU/L, >80 versus ≤80	1.667	0.597–4.655	0.330			
AST, IU/L, >80 versus ≤80	2.087	0.669–6.509	0.205			
GGT, IU/L, >64 versus ≤64	2.110	0.938–4.749	**0.071**	3.477	1.414–8.553	**0.007**
PT, second, >13 versus ≤13	2.292	1.020–5.151	**0.045**			
PLT, ×10^9^/L, <100 versus ≥100	1.751	0.733–4.182	0.207			
Child–Pugh grade, B versus A	2.614	0.914–7.472	**0.073**			
MELD score classification, >10 versus 9−10 versus <9	1.445	0.794–2.630	0.229			
CSPH, Yes versus No	2.316	1.049–5.116	**0.038**	2.711	1.160–6.335	**0.021**
Intraoperative blood loss, Yes versus No	3.088	1.339–7.121	**0.008**	3.427	1.356–8.622	**0.009**
Hepatectomy, ≥1 segment versus wedge resection	1.453	0.611–3.456	0.397			
Pringle maneuver, Yes versus No	0.444	0.190–1.036	**0.060**			
Operation time, hour, >3.0 versus ≤3.0	0.685	0.155–3.018	0.617			
Tumor diameter, cm, ≥5 versus <5	1.589	0.706–3.574	0.263			
Tumor number, Multiple versus Solitary	2.104	0.923–4.798	**0.077**	2.543	1.034–6.230	**0.042**
Microvascular invasion, Yes versus No	1.571	0.604–4.088	0.354			
Direct invasion, Yes versus No	0.619	0.080–4.813	0.647			
Node metastasis, Yes versus No	1.515	0.613–3.743	0.368			

Abbreviations: ALB, albumin; ALT, alanine transaminase; AST, aspartate aminotransferase; CI, confidence interval; CSPH, clinically significant portal hypertension; GGT, gamma‐glutamyl transpeptidase; OR, odds ratio; MELD, model for end‐stage liver disease; PHLF, postoperative liver failure; PSM, Propensity score matching; PT, prothrombin time; TBIL, total bilirubin.

Bold values indicate statistical significance.

### 3.4 Establishing the predictive nomogram for PHLF

**FIGURE 2:**
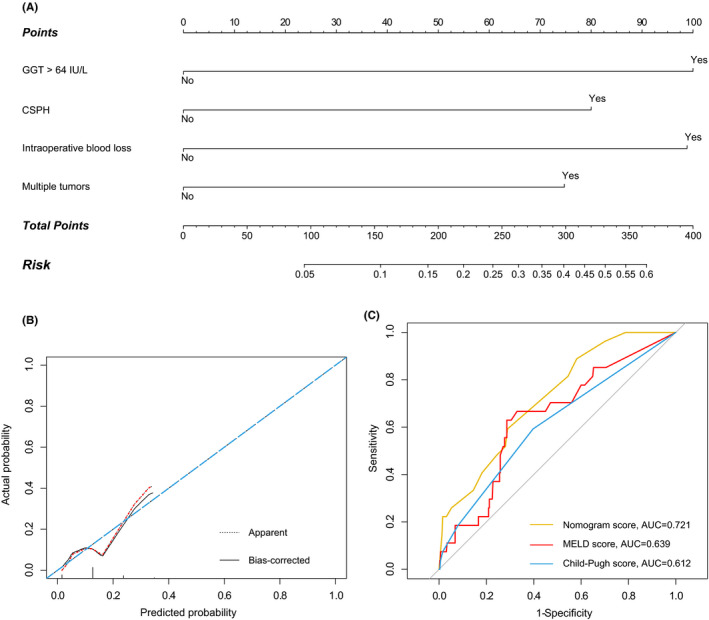
(A) The nomogram integrating GGT, CSPH, intraoperative blood loss, and multiple tumors. (B) Calibration curves of the nomogram. (C) Comparison of PHLF prediction abilities among the nomogram, MELD score, and Child–Pugh score through the ROC analysis; the nomogram has the largest AUC value

The nomogram of the PHLF probabilistic prediction model was constructed (Figure 2A) using the four factors mentioned in Table 3. The points of each factor were calculated by their weight of ORs. Then, the final score was calculated to acquire the probability of PHLF in a patient with ICC after surgery. In addition, the calibration curve showed good agreement between the likelihood of our PHLF nomogram and the actual observed incidence of PHLF in patients with ICC (Figure 2B).

Using the nomogram, we calculated the nomogram score for every patients and then compared it with the Child–Pugh and MELD scores to assess the performance of PHLF prediction through the ROC curves. Figure 2C shows that the AUC value of the nomogram score was 0.721 (95% CI: 0.630–0.812), which was greater than that of the MELD score (0.639, 95% CI: 0.532–0.747) and the Child–Pugh score (0.612, 95% CI: 0.506–0.719).

### 3.5 Impact of CSPH on long‐term prognosis in entire cohort

After follow‐up, 352 patients had a median survival of 51.9 months (IQR, 39.1, 85.5 months). Tumor recurrence occurred in 236 (67.0%) patients and 218 (61.9%) patients died.

**FIGURE 3:**
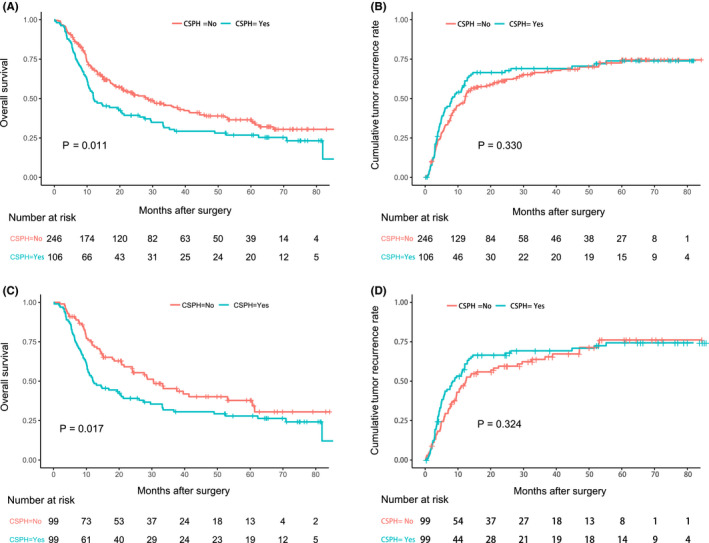
Kaplan–Meier curves for ICC patients. (A) The OS of patients with or without CSPH before PSM. (B) The TTR of patients with or without CSPH before PSM. (C) The OS of patients with or without CSPH after PSM. (D) The TTR of patients with or without CSPH after PSM

The patients without CSPH had better OS than the patients with CSPH, with 1‐, 3‐, and 5‐year OS rates of 68.0%, 45.0%, and 36.2% versus 50.0%, 30.4%, and 26.8%, respectively (*p *= 0.011, Figure 3A). However, no significant difference in tumor recurrence was found between the two groups. The 1‐, 3‐, and 5‐year recurrence rates were 47.5%, 66.8%, and 73.7% versus 57.6%, 69.3%, and 74.0%, respectively (*p *= 0.330; Figure 3B).

Table S1 shows the results of univariable analysis of OS and tumor recurrence. Multivariable analyses identified that the presence of CSPH (hazard ratio [HR]: 1.526, 95% CI: 1.148–2.027), large tumor diameter (1.061, 1.021–1.103), multiple tumors (1.569, 1.150–2.141), presence of microvascular invasion (MVI) (2.135, 1.510–3.018), and nodal metastasis (1.467, 1.059–2.030) were independent risk factors for OS. Moreover, elevated levels of CEA (1.002, 1.000–1.004), large tumor diameter (1.038, 1.011–1.078), multiple tumors (1.359, 1.005–1.837), and the presence of MVI (2.059, 1.469–2.886) were independent risk factors for tumor recurrence (Table 4).

**TABLE 4:** Multivariable Cox regression analyses of OS and tumor recurrence in the whole cohort

Variable	OS			Tumor recurrence		
	HR	95% CI	*p* value	HR	95% CI	*p* value
CEA, μg/L				1.002	1.000–1.004	**0.023**
CSPH, Yes versus No	1.526	1.148–2.027	**0.004**			
Tumor diameter, cm	1.061	1.021–1.103	**0.003**	1.038	1.011–1.078	**0.049**
Tumor number, Multiple versus Solitary	1.569	1.150–2.141	**0.005**	1.359	1.005–1.837	**0.046**
Microvascular invasion, Yes versus No	2.135	1.510–3.018	**<0.001**	2.059	1.469–2.886	**<0.001**
Node metastasis, Yes versus No	1.467	1.059–2.030	**0.021**			

Abbreviations: CA19‐9, carbohydrate antigen 19‐9; CEA, carcinoembryonic antigen; CI, confidence interval; CSPH, clinically significant portal hypertension; OS, overall survival; HR, hazard ratio.

Bold values indicate statistical significance.

### 3.6 Impact of CSPH on long‐term prognosis in the PSM cohort

Of the 198 patients of the matching cohort, 134 (67.6%) patients exhibited tumor recurrence and 126 (63.6%) patients died.

The patients with CSPH still had a worse OS than the patients without CSPH. The 1‐, 3‐, and 5‐year survival rates for these two groups were 50.5%, 31.7%, and 27.8% versus 72.9%, 45.4%, and 37.5%, respectively (*p *= 0.017; Figure 3C). No significant difference in tumor recurrence rates was found among the PSM cohort. The 1‐, 3‐, and 5‐year recurrence rates were 57.3%, 69.7%, and 74.7% versus 47.4%, 64.0%, and 76.0%, respectively, for the CSPH and non‐CSPH groups (*p *= 0.324; Figure 3D).

Table S2 shows the results of univariable analysis of OS and tumor recurrence in the PSM cohort. Multivariable analyses showed that elevated CEA levels (HR: 1.003, 95% CI: 1.000–1.006), the presence of CSPH (1.585, 1.107–2.269), large tumor diameter (1.068, 1.020–1.118), and presence of MVI (1.646, 1.022–2.648) were independent risk factors for OS. In addition, large tumor diameter (1.066, 1.019–1.115) and presence of MVI (1.062, 1.011–2.537) were independent risk factors for tumor recurrence (Table 5).

**TABLE 5:** Multivariable Cox regression analyses of OS and tumor recurrence in the PSM cohort

Variable	OS			Tumor recurrence		
	HR	95% CI	*p* value	HR	95% CI	*p* value
CEA, μg/L	1.003	1.000–1.006	**0.042**			
CSPH, Yes versus No	1.585	1.107–2.269	**0.012**			
Tumor diameter, cm	1.068	1.020–1.118	**0.005**	1.066	1.019–1.115	**0.006**
Microvascular invasion, Yes versus No	1.646	1.022–2.648	**0.040**	1.602	1.011–2.537	**0.045**

Abbreviations: CEA, carcinoembryonic antigen; CI, confidence interval; CSPH, clinically significant portal hypertension; HR, hazard ratio; OS, overall survival.

Bold values indicate statistical significance.

### 3.7 Subgroup analysis in the PSM cohort

We divided the PSM cohort into two subgroups: group tumor diameter ≥5 cm and group tumor diameter <5 cm. Kaplan–Meier curve showed that the OS significantly improved in the non‐CSPH group compared with the CSPH group with a tumor diameter ≥5 cm (*p *= 0.048). However, in patients with a diameter <5 cm (*p *= 0.141), no significant difference in OS was found between the two groups (Figure S1).

## 4 DISCUSSION

In this study, we identified CSPH and other factors significantly associated with PHLF in resectable ICC with cirrhosis and then developed a nomogram to predict postsurgical PHLF. We also analyzed the influence of CSPH on long‐term survival outcomes after LR. Analysis following PSM showed that CSPH was a significant risk factor for OS after hepatectomy, although it had no impact on recurrence.

Currently, the availability of LR for the treatment of hepatocellular carcinoma (HCC) patients with CSPH remains controversial.^26^ In accordance with the guidelines of the American Association for the Study of Liver Diseases/European Association for the Study of the Liver,^27^, ^28^ portal hypertension is considered a contraindication to LR in patients with HCC. This opinion has been supported by several regional studies over the past few years, showing that patients with HCC who are indirectly diagnosed^29^, ^30^ with portal hypertension or directly diagnosed^31^, ^32^ by hepatic venous pressure gradient (HVPG) frequently suffer from severe PHLF and poor long‐term prognosis. However, several studies from different countries reported no difference in short‐ and long‐term outcomes between portal hypertension and normal HCC patients.^33^, ^34^

In 2009, Cucchetti et al. retrospectively analyzed and compared the postoperative results and long‐term survival between 89 HCC patients with CSPH and 152 HCC patients with non‐CSPH. After PSM, the patients with CSPH had the same intraoperative course, the incidence of postoperative liver failure, morbidity, and survival rates as the patients with non‐CSPH.^33^ Santambrogio et al. divided 223 HCC patients with BCLC stage A into two groups according to the presence (*n* = 63) or absence (*n* = 160) of portal hypertension and compared the prognosis of the two groups. They reported that the short‐ and long‐term outcomes in the patients with portal hypertension were similar to those in patients with normal portal venous pressure.^29^

At present, the number of studies on short‐ and long‐term prognosis of ICC patients combined with CSPH after surgery is still limited as compared with those in HCC. Studies of ICC have mostly focused on the role of cirrhosis in the prognosis of patients after surgery.^6^, ^7^, ^35^, ^36^ Clinically, the safety and receptivity of ICC patients undergoing surgical treatment should be seriously considered in the context of CSPH. However, the impact of CSPH on postoperative outcomes, especially PHLF, has never been reported in patients with ICC. Therefore, our study is necessary and meaningful for revealing the impact of CSPH on short‐ and long‐term prognosis in ICC patients with cirrhosis after hepatectomy.

Cucchetti et al. and He et al. reported that CSPH is not an independent risk factor for OS after PSM matching.^33^, ^34^ In contrast, our study showed that the CSPH group had significantly worse OS than the non‐CSPH group, which was an independent prognostic hazard of OS after multivariable Cox regression analyses. Moreover, in the tumor diameter ≥5 cm group, subgroup analysis showed that the CSPH group had worse OS than that of the non‐CSPH group. Similarly, Zheng et al. analyzed the survival outcomes of 355 patients with HCC and found that the long‐term survival rate of patients without portal hypertension was significantly better than that of patients with portal hypertension in the cirrhosis subgroup.^30^ In addition, Berzigotti et al. reviewed 11 studies and performed a meta‐analysis in 2015, concluding that the presence of CSPH negatively affects postoperative outcomes in patients with compensatory cirrhosis who undergo surgery for HCC.^20^ Several reasons might explain this discrepancy. First, the definitions of CSPH or portal hypertension before surgery used in the aforementioned studies were different. Second, the number of patients and study periods in different articles were heterogeneous. Third, the assessment criteria of patients’ liver function and prognosis were different and not comparable.

PHLF is a serious complication of LR. The estimation and PHLF prediction could be important before treatment in cirrhotic patients. In this study, the risk of PHLF depended on several preoperative and intraoperative events, for example, GGT level, CSPH, intraoperative blood loss, and tumor numbers. GGT level was a preoperative indicator of liver function.

Recently, some studies have reported that impaired preoperative indicators, such as GGT level, platelet count, and bilirubin, are important predictors of impaired postoperative liver function.^37^, ^38^ In 2017, Hu et al.^39^ reported that high serum GGT level is an important risk factor in predicting liver failure risk after hepatectomy for patients with HCC, which is consistent with our results. We found that GGT >65 IU/ml increases the risk of PHLF after surgery in cirrhotic ICC in our PHLF prediction model.

The effect of portal hypertension on postoperative complications and PHLF in HCC is controversial.^40^ Previous studies on HCC have also verified that portal hypertension is an important determinant of PHLF.^30^, ^41^ However, no studies have revealed the impact of CSPH on PHLF in postoperative patients with ICC. Therefore, the present study is the first to reveal that CSPH is an important risk factor of PHLF. In addition, patients with ICC in the CSPH group had a significantly higher chance of overall complications than those in the non‐CSPH group.

More and more findings are suggesting that intraoperative blood loss is highly correlated with the occurrence of PHLF.^42^, ^43^ Researchers have found that intraoperative blood loss can increase the probability of ischemic reperfusion injury, leading to morbidity and mortality after liver surgery.^44^, ^45^ In addition, transfusion may induce immunosuppressive effects, which are harmful and may lead to poor perioperative outcomes in cancer patients undergoing surgery.^46^, ^47^ These studies may provide clues to explain the relationship between blood loss and PHLF. However, further research is needed to elucidate this mechanism.

Multiple tumors indicate that patients will undergo a longer surgery time and that more normal liver tissues will be removed during surgery. A sufficient future liver remnant (FLR) volume guarantees that severe postoperative complications do not occur in liver surgery.^48^ In the present study, the cirrhotic ICC patients with multiple tumors had a higher risk of PHLF after surgery compared with their counterparts.

Our study may serve as a practical guide for the surgical treatment of patients with CSPH in the future. This study can also be used to select suitable patients for surgery. On the one hand, CSPH patients who meet the proper surgical requirements will not only benefit from a good prognosis, but will also benefit from a reduced occurrence of PHLF. Many factors determine whether or not patients with CSPH can receive surgical treatment, and these need further exploration by researchers. This is only a retrospective study from a single‐center, and more global multicenter data or prospective studies are needed to verify our research conclusions or generate new ideas. On the other hand, surgeons must pay attention to CSPH because of its significant impact on the perioperative period and prognosis. More and more new discoveries are expected in the future as the understanding of CSPH is deepened. In the next 5 or even 10 years, from the aspect of surgical operation, more unresectable CSPH patients can benefit from continuous technological progress in surgical techniques, advanced medical equipment (e.g., laparoscopic and robotic surgery), and virtual reality technology applied in precision LR. From the aspect of comprehensive treatment, with the rapid development of the system therapy in liver cancer, immunotherapy and targeted therapy system has become the main treatment of advanced liver cancer. These treatment methods enrich the options for advanced liver cancer, especially for patients with CSPH. Patients with multiple tumors and tumors larger than 5 cm may benefit from neoadjuvant therapy and sequential surgical treatment. This finding needs to be confirmed by further clinical studies.

Our study has several limitations. First, this is a single‐institution retrospective study. More patients from more institutions need to be studied to confirm our conclusions. Second, ICG‐R15 and FLR are important indicators used to assess liver function and volume in clinical practice. However, in this study, these data were missing for various reasons, resulting in an imperfect study. These two variables must be added in our future studies. Third, CSPH was diagnosed indirectly by clinical criteria; thus, we could not determine the real influence of HVPG measurement as a diagnostic criterion for ICC patients with CSPH. Last, given the limitations of the PSM approach, our study still had the potential for selection bias, although we used PSM in an attempt to decrease these biases.

## 5 CONCLUSION

The presence of CSPH is significantly associated with PHLF and a worse OS in patients with ICC after hepatectomy. CSPH patients with high levels of CEA, GGT, multiple tumors, and large tumor diameter (≥5 cm) preoperatively are not recommended to undergo surgical treatment to avoid postoperative liver failure and poor prognosis.

## ETHICS STATEMENT

The study protocol was performed in accordance with the guidelines outlined in the Declaration of Helsinki. The Institutional Review Board of Mengchao Hepatobiliary Hospital of Fujian Medical University approved the study (ID: 2018‐048‐01). The patients/participants provided their written informed consent.

## CONFLICT OF INTEREST

The authors declare that the research was conducted in the absence of any commercial or financial relationships that could be construed as a potential conflict of interest.

## Supporting information

Supplementary Material

## Data Availability

The data involved in this study are available upon request to the corresponding author.

## References

[1] Chang KY , Chang JY , Yen Y . Increasing incidence of intrahepatic cholangiocarcinoma and its relationship to chronic viral hepatitis. J Natl Compr Canc Netw. 2009;7(4):423‐427. 19406042 10.6004/jnccn.2009.0030

[2] Amini N , Ejaz A , Spolverato G , Kim Y , Herman JM , Pawlik TM . Temporal trends in liver‐directed therapy of patients with intrahepatic cholangiocarcinoma in the United States: a population‐based analysis. J Surg Oncol. 2014;110(2):163‐170. 24676600 10.1002/jso.23605

[3] Rizvi S , Gores GJ . Pathogenesis, diagnosis, and management of cholangiocarcinoma. Gastroenterology. 2013;145(6):1215‐1229. 24140396 10.1053/j.gastro.2013.10.013 PMC3862291

[4] Razumilava N , Gores GJ . Cholangiocarcinoma. Lancet. 2014;383(9935):2168‐2179. 24581682 10.1016/S0140-6736(13)61903-0 PMC4069226

[5] Wu Z‐F , Wu X‐Y , Zhu N , et al. Prognosis after resection for hepatitis B virus‐associated intrahepatic cholangiocarcinoma. World J Gastroenterol. 2015;21(3):935‐943. 25624728 10.3748/wjg.v21.i3.935 PMC4299347

[6] Li Y‐Y , Li H , Lv P , et al. Prognostic value of cirrhosis for intrahepatic cholangiocarcinoma after surgical treatment. J Gastrointest Surg. 2011;15(4):608‐613. 21246412 10.1007/s11605-011-1419-8

[7] Li H , Wu J‐S , Wang X‐T , et al. Major hepatectomy is a safe modality for the treatment of intrahepatic cholangiocarcinoma in selected patients complicated with cirrhosis. J Gastrointest Surg. 2014;18(1):194‐199. 24222320 10.1007/s11605-013-2363-6

[8] Tsochatzis EA , Bosch J , Burroughs AK . Liver cirrhosis. Lancet (London, England). 2014;383(9930):1749‐1761. 10.1016/S0140-6736(14)60121-5 24480518

[9] Bridgewater J , Galle PR , Khan SA , et al. Guidelines for the diagnosis and management of intrahepatic cholangiocarcinoma. J Hepatol. 2014;60(6):1268‐1289. 24681130 10.1016/j.jhep.2014.01.021

[10] Cillo U , Fondevila C , Donadon M , et al. Surgery for cholangiocarcinoma. Liver Int. 2019;39(suppl 1):143–155. 30843343 10.1111/liv.14089 PMC6563077

[11] Hemming AW , Reed AI , Fujita S , Foley DP , Howard RJ . Surgical management of hilar cholangiocarcinoma. Ann Surg. 2005;241(5):693–699; discussion 9–702. 15849505 10.1097/01.sla.0000160701.38945.82 PMC1357124

[12] Farges O , Fuks D . Clinical presentation and management of intrahepatic cholangiocarcinoma. Gastroenterol Clin Biol. 2010;34(3):191‐199. 20202770 10.1016/j.gcb.2010.01.006

[13] Hyder O , Hatzaras I , Sotiropoulos GC , et al. Recurrence after operative management of intrahepatic cholangiocarcinoma. Surgery. 2013;153(6):811‐818. 23499016 10.1016/j.surg.2012.12.005 PMC3980567

[14] Farges O , Fuks D , Boleslawski E , et al. Influence of surgical margins on outcome in patients with intrahepatic cholangiocarcinoma: a multicenter study by the AFC‐IHCC‐2009 study group. Ann Surg. 2011;254(5):824‐829. discussion 30. 22042474 10.1097/SLA.0b013e318236c21d

[15] de Jong MC , Nathan H , Sotiropoulos GC , et al. Intrahepatic cholangiocarcinoma: an international multi‐institutional analysis of prognostic factors and lymph node assessment. J Clin Oncol. 2011;29(23):3140‐3145. 21730269 10.1200/JCO.2011.35.6519

[16] Maithel SK , Gamblin TC , Kamel I , Corona‐Villalobos CP , Thomas M , Pawlik TM . Multidisciplinary approaches to intrahepatic cholangiocarcinoma. Cancer. 2013;119(22):3929‐3942. 23963845 10.1002/cncr.28312

[17] Rizzo A , Ricci AD , Brandi G . Futibatinib, an investigational agent for the treatment of intrahepatic cholangiocarcinoma: evidence to date and future perspectives. Expert Opin Investig Drugs. 2020;30:317–324. 10.1080/13543784.2021.1837774 33054456

[18] Chen Q , Li F , Gao Y , et al. Developing a selection‐aided model to screen cirrhotic intrahepatic cholangiocarcinoma for hepatectomy. J Cancer. 2020;11(19):5623‐5634. 32913457 10.7150/jca.46587 PMC7477447

[19] Wang Y , Li J , Xia Y , et al. Prognostic nomogram for intrahepatic cholangiocarcinoma after partial hepatectomy. J Clin Oncol. 2013;31(9):1188‐1195. 23358969 10.1200/JCO.2012.41.5984

[20] Berzigotti A , Reig M , Abraldes JG , Bosch J , Bruix J . Portal hypertension and the outcome of surgery for hepatocellular carcinoma in compensated cirrhosis: a systematic review and meta‐analysis. Hepatology. 2015;61(2):526‐536. 25212123 10.1002/hep.27431

[21] Freeman RB Jr , Wiesner RH , Harper A , et al. The new liver allocation system: moving toward evidence‐based transplantation policy. Liver Transpl. 2002;8(9):851‐858. 12200791 10.1053/jlts.2002.35927

[22] Allard MA , Adam R , Bucur PO , et al. Posthepatectomy portal vein pressure predicts liver failure and mortality after major liver resection on noncirrhotic liver. Ann Surg. 2013;258(5):822–829; discussion 9–30. 24045452 10.1097/SLA.0b013e3182a64b38

[23] Dindo D , Demartines N , Clavien PA . Classification of surgical complications: a new proposal with evaluation in a cohort of 6336 patients and results of a survey. Ann Surg. 2004;240(2):205‐213. 15273542 10.1097/01.sla.0000133083.54934.ae PMC1360123

[24] Stuart EA . Matching methods for causal inference: a review and a look forward. Statistical Sci. 2010;25(1):1‐21. 10.1214/09-STS313 PMC2943670 20871802

[25] D'Agostino RB Jr . Propensity score methods for bias reduction in the comparison of a treatment to a non‐randomized control group. Stat Med. 1998;17(19):2265‐2281. 9802183 10.1002/(sici)1097-0258(19981015)17:19<2265::aid-sim918>3.0.co;2-b

[26] Liu J , Zhang H , Xia Y , et al. Impact of clinically significant portal hypertension on outcomes after partial hepatectomy for hepatocellular carcinoma: a systematic review and meta‐analysis. HPB (Oxford). 2019;21(1):1‐13. 30082213 10.1016/j.hpb.2018.07.005

[27] Bruix J , Sherman M , Practice Guidelines Committee AAftSoLD . Management of hepatocellular carcinoma. Hepatology. 2005;42(5):1208‐1236. 16250051 10.1002/hep.20933

[28] Bruix J , Sherman M , Llovet JM , et al. Clinical management of hepatocellular carcinoma. Conclusions of the Barcelona‐2000 EASL conference. European Association for the Study of the Liver. J Hepatol. 2001;35(3):421‐430. 11592607 10.1016/s0168-8278(01)00130-1

[29] Santambrogio R , Kluger MD , Costa M , et al. Hepatic resection for hepatocellular carcinoma in patients with Child‐Pugh's A cirrhosis: is clinical evidence of portal hypertension a contraindication? HPB (Oxford). 2013;15(1):78‐84. 23216782 10.1111/j.1477-2574.2012.00594.x PMC3533715

[30] Zheng Y‐W , Wang K‐P , Zhou J‐J , et al. Portal hypertension predicts short‐term and long‐term outcomes after hepatectomy in hepatocellular carcinoma patients. Scand J Gastroenterol. 2018;53(12):1562‐1568. 30572742 10.1080/00365521.2018.1538386

[31] Boleslawski E , Petrovai G , Truant S , et al. Hepatic venous pressure gradient in the assessment of portal hypertension before liver resection in patients with cirrhosis. Br J Surg. 2012;99(6):855‐863. 22508371 10.1002/bjs.8753

[32] Stremitzer S , Tamandl D , Kaczirek K , et al. Value of hepatic venous pressure gradient measurement before liver resection for hepatocellular carcinoma. Br J Surg. 2011;98(12):1752‐1758. 22009385 10.1002/bjs.7672

[33] Cucchetti A , Ercolani G , Vivarelli M , et al. Is portal hypertension a contraindication to hepatic resection? Ann Surg. 2009;250(6):922‐928. 19855258 10.1097/SLA.0b013e3181b977a5

[34] He W , Zeng Q , Zheng Y , et al. The role of clinically significant portal hypertension in hepatic resection for hepatocellular carcinoma patients: a propensity score matching analysis. BMC Cancer. 2015;15:263. 25886495 10.1186/s12885-015-1280-3 PMC4399206

[35] Peng N‐F , Li L‐Q , Qin X , et al. Evaluation of risk factors and clinicopathologic features for intrahepatic cholangiocarcinoma in Southern China: a possible role of hepatitis B virus. Ann Surg Oncol. 2011;18(5):1258‐1266. 21207172 10.1245/s10434-010-1458-5

[36] Jeong S , Gao L , Tong Y , et al. Prognostic impact of cirrhosis in patients with intrahepatic cholangiocarcinoma following hepatic resection. Can J Gastroenterol Hepatol. 2017; 10.1155/2017/6543423 PMC5702404 29259967

[37] Kubota K , Makuuchi M , Kusaka K , et al. Measurement of liver volume and hepatic functional reserve as a guide to decision‐making in resectional surgery for hepatic tumors. Hepatology. 1997;26(5):1176‐1181. 9362359 10.1053/jhep.1997.v26.pm0009362359

[38] Yang T , Zhang J , Lu JH , Yang GS , Wu MC , Yu WF . Risk factors influencing postoperative outcomes of major hepatic resection of hepatocellular carcinoma for patients with underlying liver diseases. World J Surg. 2011;35(9):2073‐2082. 21656309 10.1007/s00268-011-1161-0

[39] Hu H , Han H , Han XK , Wang WP , Ding H . Nomogram for individualised prediction of liver failure risk after hepatectomy in patients with resectable hepatocellular carcinoma: the evidence from ultrasound data. Eur Radiol. 2018;28(2):877‐885. 28779402 10.1007/s00330-017-4900-2

[40] Hackl C , Schlitt HJ , Renner P , Lang SA . Liver surgery in cirrhosis and portal hypertension. World J Gastroenterol. 2016;22(9):2725‐2735. 26973411 10.3748/wjg.v22.i9.2725 PMC4777995

[41] Zou H , Wen Y , Yuan K , Miao XY , Xiong L , Liu KJ . Combining albumin‐bilirubin score with future liver remnant predicts post‐hepatectomy liver failure in HBV‐associated HCC patients. Liver Int. 2018;38(3):494‐502. 28685924 10.1111/liv.13514

[42] Lee EC , Park S‐J , Han S‐S , et al. Risk prediction of post‐hepatectomy liver failure in patients with perihilar cholangiocarcinoma. J Gastroenterol Hepatol. 2018;33(4):958‐965. 28843035 10.1111/jgh.13966

[43] Van Den Broek MAJ , Olde Damink SWM , Dejong CHC , et al. Liver failure after partial hepatic resection: definition, pathophysiology, risk factors and treatment. Liver Int. 2008;28(6):767‐780. 18647141 10.1111/j.1478-3231.2008.01777.x

[44] Ezaki T , Seo Y , Tomoda H , Furusawa M , Kanematsu T , Sugimachi K . Partial hepatic resection under intermittent hepatic inflow occlusion in patients with chronic liver disease. Br J Surg. 1992;79(3):224‐226. 1313324 10.1002/bjs.1800790311

[45] Glanemann M , Langrehr JM , Stange BJ , et al. Clinical implications of hepatic preservation injury after adult liver transplantation. Am J Transplant. 2003;3(8):1003‐1009. 12859537 10.1034/j.1600-6143.2003.00167.x

[46] Kwon AH , Matsui Y , Kamiyama Y . Perioperative blood transfusion in hepatocellular carcinomas: influence of immunologic profile and recurrence free survival. Cancer. 2001;91(4):771‐778. 11241245

[47] Kaplan J , Sarnaik S , Gitlin J , Lusher J . Diminished helper/suppressor lymphocyte ratios and natural killer activity in recipients of repeated blood transfusions. Blood. 1984;64(1):308‐310. 6234037

[48] Kishi Y , Abdalla EK , Chun YS , et al. Three hundred and one consecutive extended right hepatectomies: evaluation of outcome based on systematic liver volumetry. Ann Surg. 2009;250(4):540‐548. 19730239 10.1097/SLA.0b013e3181b674df

