# Supplementary material for: Impact of portal hypertension on short‐ and long‐term outcomes after liver resection for intrahepatic cholangiocarcinoma: A propensity score matching analysis
Source: Cancer Med. 2021 Aug 18;10(20):6985–97. doi: 10.1002/cam4.4222 (PMC8525133; doi:10.1002/cam4.4222)
Supplement: Supplementary file 1 — Supplementary Material [file CAM4-10-6985-s001.pdf]

Content of supplementary files:

Supplementary Figure S1 Kaplan–Meier curves for ICC patients in the PSM cohort stratified based on tumor diameter. (A) The OS of the patients with or without CSPH in group tumor diameter < 5 cm. (B) The TTR of the patients with or without CSPH in group tumor diameter < 5 cm. (C) The OS of the patients with or without CSPH in group tumor diameter ≥ 5 cm. (D) The TTR of the patients with or without CSPH in group tumor diameter ≥ 5 cm.

Supplementary Table S1 Univariable Cox regression analyses of OS and tumor recurrence in the whole cohort.

Supplementary Table S2 Univariable Cox regression analyses of OS and tumor recurrence in the PSM cohort.

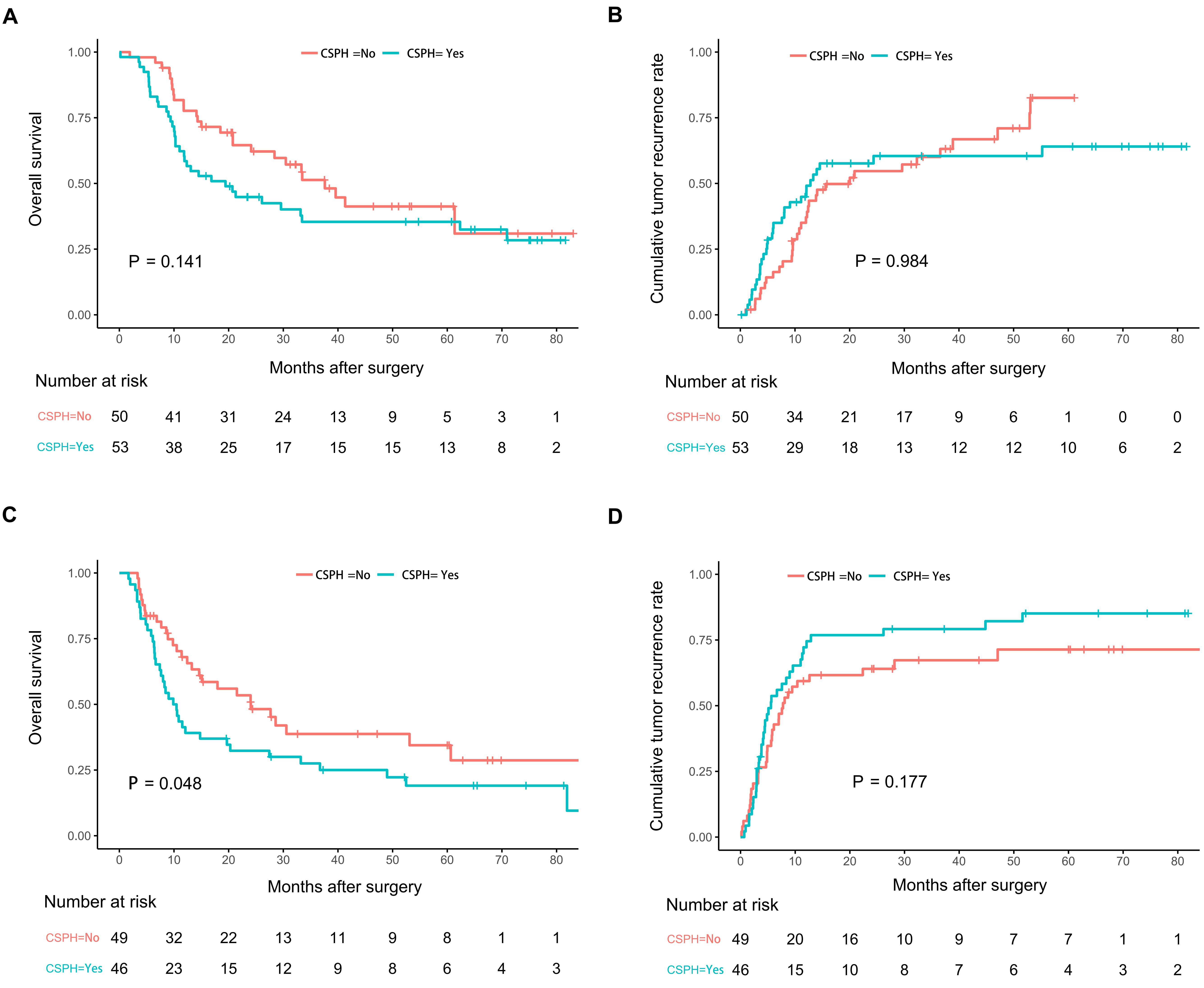
A
Overall survival
1.00
0.75
0.50
0.25
0.00
CSPH =No
CSPH= Yes
P = 0.141
0 10 20 30 40 50 60 70 80
Months after surgery
Number at risk
CSPH=No 50 41 31 24 13 9 5 3 1
CSPH=Yes 53 38 25 17 15 15 13 8 2
B
Cumulative tumor recurrence rate
1.00
0.75
0.50
0.25
0.00
CSPH =No
CSPH= Yes
P = 0.984
0 10 20 30 40 50 60 70 80
Months after surgery
Number at risk
CSPH=No 50 34 21 17 9 6 1 0 0
CSPH=Yes 53 29 18 13 12 12 10 6 2
C
Overall survival
1.00
0.75
0.50
0.25
0.00
CSPH =No
CSPH= Yes
P = 0.048
0 10 20 30 40 50 60 70 80
Months after surgery
Number at risk
CSPH=No 49 32 22 13 11 9 8 1 1
CSPH=Yes 46 23 15 12 9 8 6 4 3
D
Cumulative tumor recurrence rate
1.00
0.75
0.50
0.25
0.00
CSPH =No
CSPH= Yes
P = 0.177
0 10 20 30 40 50 60 70 80
Months after surgery
Number at risk
CSPH=No 49 20 16 10 9 7 7 1 1
CSPH=Yes 46 15 10 8 7 6 4 3 2

Supplementary Table S1 Univariable Cox regression analyses of OS and tumor recurrence in the whole cohort.

Variable	OS			Tumor recurrence		
	HR	95% CI	*P* value	HR	95%CI	*P* value
**Age**, Year	1.002	0.989 - 1.014	0.768	1.002	0.991 - 1.015	0.684
**Age**, Year, > 65 vs. ≤ 65	0.956	0.639 - 1.431	0.827	1.049	0.723 - 1.523	0.800
**Gender**, Male vs. Female	1.217	0.823 - 1.800	0.324	1.219	0.843 - 1.762	0.291
**BMI**	0.977	0.944 - 1.011	0.180	0.982	0.95 - 1.015	0.275
**Hepatobiliary flukes**, Yes vs. No	0.393	0.098 - 1.582	0.173	0.557	0.178 - 1.741	0.307
**Hepatolithiasis**, Yes vs. No	1.022	0.604 - 1.727	0.936	0.969	0.591 - 1.587	0.899
**HBsAg**, Positive vs. Negative	0.898	0.625 - 1.290	0.559	0.913	0.645 - 1.292	0.608
**HCV-Ab**, Positive vs. Negative	0.527	0.169 - 1.649	0.263	0.292	0.073 - 1.175	0.065
**TBIL**, mg/dL	1.023	0.979 - 1.068	0.313	0.995	0.946 - 1.045	0.833
**ALB**, g/L	0.973	0.945 - 1.002	0.070	0.986	0.959 - 1.013	0.301
**ALT**, IU/L	0.999	0.997 - 1.002	0.501	0.999	0.997 - 1.002	0.361
**AST**, IU/L	1.000	0.997 - 1.003	0.335	1.001	0.998 - 1.003	0.149
**GGT**, IU/L	1.000	1.000 - 1.001	0.977	1.000	1.000 - 1.001	0.902

**PT**, Second	1.083	0.975 - 1.202	0.139	1.091	0.977 - 1.218	0.124
**PLT**, ×10^9/L	1.001	0.999 - 1.004	0.191	1.001	0.999 - 1.003	0.500
**Child-Pugh score**	1.094	0.928 - 1.289	0.285	0.970	0.816 - 1.153	0.730
**Child-Pugh grade**, B vs. A	1.243	0.799 - 1.932	0.333	0.999	0.632 - 1.579	0.995
**MELD score**	1.040	0.982 - 1.101	0.178	1.010	0.951 - 1.072	0.757
**MELD score**						
9−10 vs. < 9	0.768	0.338 - 1.745	0.528	0.480	0.177- 1.300	0.149
> 10 vs. < 9	0.776	0.362 - 1.666	0.516	0.578	0.254 - 1.314	0.191
**AFP**, μg/L	1.000	1.000 - 1.001	0.491	1.000	1.000 - 1.001	0.107
**CEA**, μg/L	1.001	1.000 - 1.003	**0.043**	1.003	1.001 - 1.005	**<0.001**
**CA19-9**, IU/mL	1.001	1.000 - 1.001	**0.001**	1.001	1.000 - 1.001	**0.029**
**CSPH**, Yes vs. No	1.433	1.086 - 1.892	**0.011**	1.148	0.870 - 1.515	0.329
**Intraoperative blood loss**, Yes vs. No	1.542	1.114 - 2.136	**0.009**	1.259	0.901 - 1.758	0.176
**Volume of intraoperative blood loss**, mL	1.000	1.000 - 1.000	0.060	1.000	1.000 - 1.000	**0.045**
**Hepatectomy**, ≥1 segments vs. wedge resection	1.050	0.765 - 1.440	0.763	1.034	0.765 - 1.398	0.828
**Pringle maneuver**, Yes vs. No	0.810	0.583 - 1.125	0.207	0.948	0.685 - 1.312	0.747

**Clamp time**, minute	1.000	0.986 - 1.014	0.984	1.003	0.99 - 1.017	0.632
**Operation time**, hour	1.065	0.988 - 1.148	0.101	1.046	0.969 - 1.129	0.252
**Resection margin**, cm	0.809	0.623 - 1.052	0.113	0.989	0.711 - 1.138	0.378
**Tumor diameter**, cm	1.085	1.047 - 1.123	**<0.001**	1.068	1.033 - 1.105	**<0.001**
**Tumor number**, Multiple vs. Solitary	1.615	1.199 - 2.175	**0.001**	1.483	1.107 - 1.987	**0.008**
**Microvascular invasion**, Yes vs. No	2.398	1.723 - 3.339	**<0.001**	2.253	1.633 - 3.108	**<0.001**
**Direct invasion**, Yes vs. No	2.003	1.218 - 3.296	**0.005**	1.817	1.107 - 2.981	**0.016**
**Node metastasis**, Yes vs. No	1.450	1.054 - 1.995	**0.022**	1.325	0.970 - 1.812	0.076

Abbreviations: OS, overall survival; HR, hazard ratio; CI confidence interval; BMI, Body Mass Index; HBsAg, hepatitis B surface antigen; HCV, hepatitis C virus; TBIL, total bilirubin; ALB, albumin; ALT, alanine transaminase; AST, aspartate aminotransferase; GGT, gamma-glutamyl transpeptidase; PT, prothrombin time; PLT, platelet; MELD, model for end-stage liver disease; AFP, alpha-fetoprotein; CEA, carcinoembryonic antigen; CA19-9, carbonhydrateantigen19-9; CSPH, clinically significant portal hypertension.

Supplementary Table S2 Univariable Cox regression analyses of OS and tumor recurrence in the PSM cohort.

Variable	OS			Tumor recurrence		
	HR	95% CI	*P* value	HR	95%CI	*P* value
**Age**, Year	1.001	0.984 - 1.018	0.927	1.005	0.989 - 1.021	0.548
**Age**, Year, > 65 vs. ≤ 65	1.000	0.613 - 1.632	1.000	1.108	0.701 - 1.75	0.661
**Gender**, Male vs. Female	1.003	0.609 - 1.653	0.990	1.077	0.662 - 1.752	0.764
**BMI**	0.960	0.917 - 1.006	0.085	0.962	0.919 - 1.007	0.099
**Hepatobiliary flukes**, Yes vs. No	0.198	0.028 - 1.414	0.072	0.384	0.095 - 1.554	0.163
**Hepatolithiasis**, Yes vs. No	1.518	0.817 - 2.820	0.183	1.303	0.703 - 2.415	0.400
**HBsAg**, Positive vs. Negative	1.172	0.718 - 1.912	0.525	1.042	0.659 - 1.646	0.861
**HCV-Ab**, Positive vs. Negative	0.670	0.213 - 2.108	0.491	0.359	0.089 - 1.452	0.134
**TBIL**, mg/dL	1.016	0.966 - 1.069	0.538	0.987	0.931 - 1.046	0.662
**ALB**, g/L	0.983	0.946 - 1.021	0.382	1.000	0.963 - 1.038	0.989
**ALT**, IU/L	0.999	0.997 - 1.002	0.537	1.000	0.998 - 1.002	0.901
**AST**, IU/L	0.999	0.997 - 1.002	0.630	1.001	0.999 - 1.004	0.178
**GGT**, IU/L	1.000	1.000 - 1.001	0.290	1.000	1.000 - 1.001	0.298

**PT**, Second	1.040	0.899 - 1.203	0.598	1.052	0.918 - 1.206	0.465
**PLT**, ×10^9/L	1.000	0.997 - 1.003	0.965	0.999	0.996 - 1.001	0.317
**Child-Pugh score**	1.043	0.840 - 1.294	0.705	0.933	0.746 - 1.166	0.543
**Child-Pugh grade**, B vs. A	1.133	0.624 - 2.056	0.681	0.951	0.525 - 1.721	0.868
**MELD score**	1.028	0.954 - 1.109	0.464	0.979	0.904 - 1.061	0.608
**MELD score**						
9−10 vs. < 9	1.150	0.609 - 2.171	0.667	0.617	0.274- 1.389	0.244
> 10 vs. < 9	1.055	0.624 - 1.785	0.840	0.969	0.564 - 1.665	0.910
**AFP**, µg/L	1.000	1.000 - 1.001	0.320	1.000	1.000 - 1.001	0.116
**CEA**, µg/L	1.004	1.001 - 1.006	**<0.001**	1.003	1.001 - 1.006	**0.003**
**CA19-9**, IU/mL	1.001	1.000 - 1.001	**0.015**	1.001	1.000 - 1.001	**0.026**
**CSPH**, Yes vs. No	1.532	1.075 - 2.183	**0.017**	1.186	0.845 - 1.666	0.323
**Intraoperative blood loss**, Yes vs. No	1.421	0.934 - 2.164	0.099	1.311	0.859 - 2.001	0.208
**Volume of intraoperative blood loss**, mL	1.000	1.000 - 1.000	0.422	1.000	1.000 - 1.000	0.103
**Hepatectomy**, ≥1 segments vs. wedge resection	0.991	0.635 - 1.547	0.969	1.050	0.688 - 1.602	0.820
**Pringle maneuver**, Yes vs. No	0.772	0.507 - 1.177	0.228	0.876	0.577 - 1.329	0.532

**Clamp time**, minute	0.998	0.980 - 1.017	0.865	1.000	0.983 - 1.018	0.964
**Operation time**, hour	1.070	0.914 - 1.253	0.397	1.145	0.972 - 1.349	0.105
**Resection margin**, cm	0.846	0.605 - 1.183	0.328	0.819	0.594 - 1.129	0.221
**Tumor diameter**, cm	1.080	1.034 - 1.128	**0.001**	1.073	1.028 - 1.120	**0.001**
**Tumor number**, Multiple vs. Solitary	1.395	0.864 - 2.253	0.171	1.311	0.814 - 2.111	0.264
**Microvascular invasion**, Yes vs. No	1.782	1.132 - 2.805	**0.011**	1.846	1.194 - 2.854	**0.005**
**Direct invasion**, Yes vs. No	1.949	1.044 - 3.638	**0.033**	1.754	0.945 - 3.259	0.071
**Node metastasis**, Yes vs. No	1.583	1.043 - 2.402	**0.030**	1.577	1.054 - 2.36	**0.025**

Abbreviations: OS, overall survival; HR, hazard ratio; CI confidence interval; BMI, Body Mass Index; HBsAg, hepatitis B surface antigen; HCV, hepatitis C virus; TBIL, total bilirubin; ALB, albumin; ALT, alanine transaminase; AST, aspartate aminotransferase; GGT, gamma-glutamyl transpeptidase; PT, prothrombin time; PLT, platelet; MELD, model for end-stage liver disease; AFP, alpha-fetoprotein; CEA, carcinoembryonic antigen; CA19-9, carbonhydrateantigen19-9; CSPH, clinically significant portal hypertension.
